# Geographic patterns of mtDNA and Z-linked sequence variation in the Common Chiffchaff and the ‘chiffchaff complex’

**DOI:** 10.1371/journal.pone.0210268

**Published:** 2019-01-04

**Authors:** Marko Raković, Júlio M. Neto, Ricardo J. Lopes, Evgeniy A. Koblik, Igor V. Fadeev, Yuriy V. Lohman, Sargis A. Aghayan, Giovanni Boano, Marco Pavia, Yoav Perlman, Yosef Kiat, Amir Ben Dov, J. Martin Collinson, Gary Voelker, Sergei V. Drovetski

**Affiliations:** 1 Natural History Museum Belgrade, Belgrade, Serbia; 2 Molecular Ecology and Evolution Lab, Department of Biology, Ecology Building, Lund, Sweden; 3 CIBIO, Centro de Investigação em Biodiversidade e Recursos Genéticos da Universidade do Porto, InBIO Laboratório Associado, Universidade do Porto, Vairão, Portugal; 4 Department of Ornithology, Zoological Museum of Moscow State University, Moscow, Russia; 5 Department of Collections, State Darwin Museum, Moscow, Russia; 6 Independent Researcher, Krasnodar, Russia; 7 Laboratory of Zoology, Research Institute of Biology, Yerevan State University, Yerevan, Armenia; 8 Natural History Museum of Carmagnola, Carmagnola, Italy; 9 Department of Earth Sciences, University of Turin, Turin, Italy; 10 Israeli Ornithological Centre, Society for the Protection of Nature in Israel, Tel Aviv, Israel; 11 Movement Ecology Laboratory, Department of Ecology, Evolution and Behavior, Alexander Silberman Institute of Life Sciences, Edmond J. Safra campus, The Hebrew University of Jerusalem, Jerusalem, Israel; 12 Independent Researcher, Petah Tikva, Israel; 13 Institute of Medicine, Medical Sciences and Nutrition, University of Aberdeen Foresterhill, Aberdeen, United Kingdom; 14 Department of Wildlife and Fisheries Sciences, Texas A&M University, College Station, Texas, United States of America; 15 Laboratories of Analytical Biology, National Museum of Natural History, Smithsonian Institution, Washington DC, United States of America; National Cheng Kung University, TAIWAN

## Abstract

The Common Chiffchaff *Phylloscopus collybita* is an abundant, polytypic Palearctic bird. Validity of some of its subspecies is controversial and birds from some parts of the species range remain unclassified taxonomically. The relationships among populations from different geographic areas have not been sufficiently explored with molecular data. In this study we analyzed the relationships among the four species in the ‘chiffchaff complex’ (Common Chiffchaff, Iberian Chiffchaff *P*. *ibericus*, Canary Islands Chiffchaff *P*. *canariensis* and Mountain Chiffchaff *P*. *sindianus*), and the patterns of intraspecific geographic variation in the mtDNA ND2 gene and intron 9 of the Z-linked aconitase gene (ACO1I9) across the Common Chiffchaff range, including a recently discovered population breeding on Mt. Hermon (Anti-Lebanon mountains). Our data supported the monophyly of the chiffchaff complex and its current systematics at the species level. Within the Common Chiffchaff, the Siberian race *P*. *c*. *tristis* was the most differentiated subspecies and may represent a separate or incipient species. Other Common Chiffchaff subspecies also were differentiated in their mtDNA, however, lineages of neighboring subspecies formed wide zones of introgression. The Mt. Hermon population was of mixed genetic origin but contained some birds with novel unique lineage that could not be assigned to known subspecies. All Common Chiffchaff lineages diverged at the end of the Ionian stage of Pleistocene. Lineage sorting of ACO1I9 alleles was not as complete as that of mtDNA. Chiffchaff species were mostly distinct at ACO1I9, except the Common and Canary Islands Chiffchaffs that shared multiple alleles. An AMOVA identified geographic structure in Common Chiffchaff ACO1I9 variation that was broadly consistent with that of mtDNA ND2 gene. The genetic and other data suggest the chiffchaff complex to be a group of evolutionarily young taxa that represent a paradigm of ‘species evolution in action’ from intergrading subspecies through to apparently complete biological speciation.

## Introduction

The ‘chiffchaff complex’ (Aves: Phylloscopidae) is a group of Old World Leaf Warblers in the genus *Phylloscopus*. Species from this complex (Common Chiffchaff *P*. *collybita*, Iberian Chiffchaff *P*. *ibericus*, Mountain Chiffchaff *P*. *sindianus* and Canary Islands Chiffchaff *P*. *canariensis*) historically have been considered as a single species. All of them have superficially similar, dull green/brown, relatively featureless plumages, but explosive, loud advertising songs including the characteristic repeated ‘djip djup’ song of Common Chiffchaff (and variations of this song in other species) that gives the complex its name. The Common Chiffchaff is an abundant forest bird of boreal and temperate Palearctic. Its range extends from the British Isles and Scandinavia to the Kolyma River in Eastern Siberia. The mountains of southern Eurasia, including the Pyrenees, ranges of the Apennine, Balkan, and Anatolian Peninsulas, the Caucasus, Transcaucasia, northern Iran and southwestern Turkmenistan, and southern Siberia form the southern edge of the Common Chiffchaff's range. Its northern edge coincides with the limit of arboreal vegetation [1,2]. The breeding range of Iberian Chiffchaff is restricted to the southernmost France, northern Spain, Portugal, and northwest Africa, overlapping narrowly with the southern limit of Common Chiffchaff in the Pyrenees. Mountain Chiffchaff breeds predominantly in high altitude forests in the Caucasus and Transcaucasia (subspecies *P*. *s*. *lorenzii*), overlapping with southern populations of Common Chiffchaff, and in the mountains of Central Asia (*P*. *s*. *sindianus*). Canary Islands Chiffchaff is restricted to the western Canary Islands (Spain).

**Fig 1:**
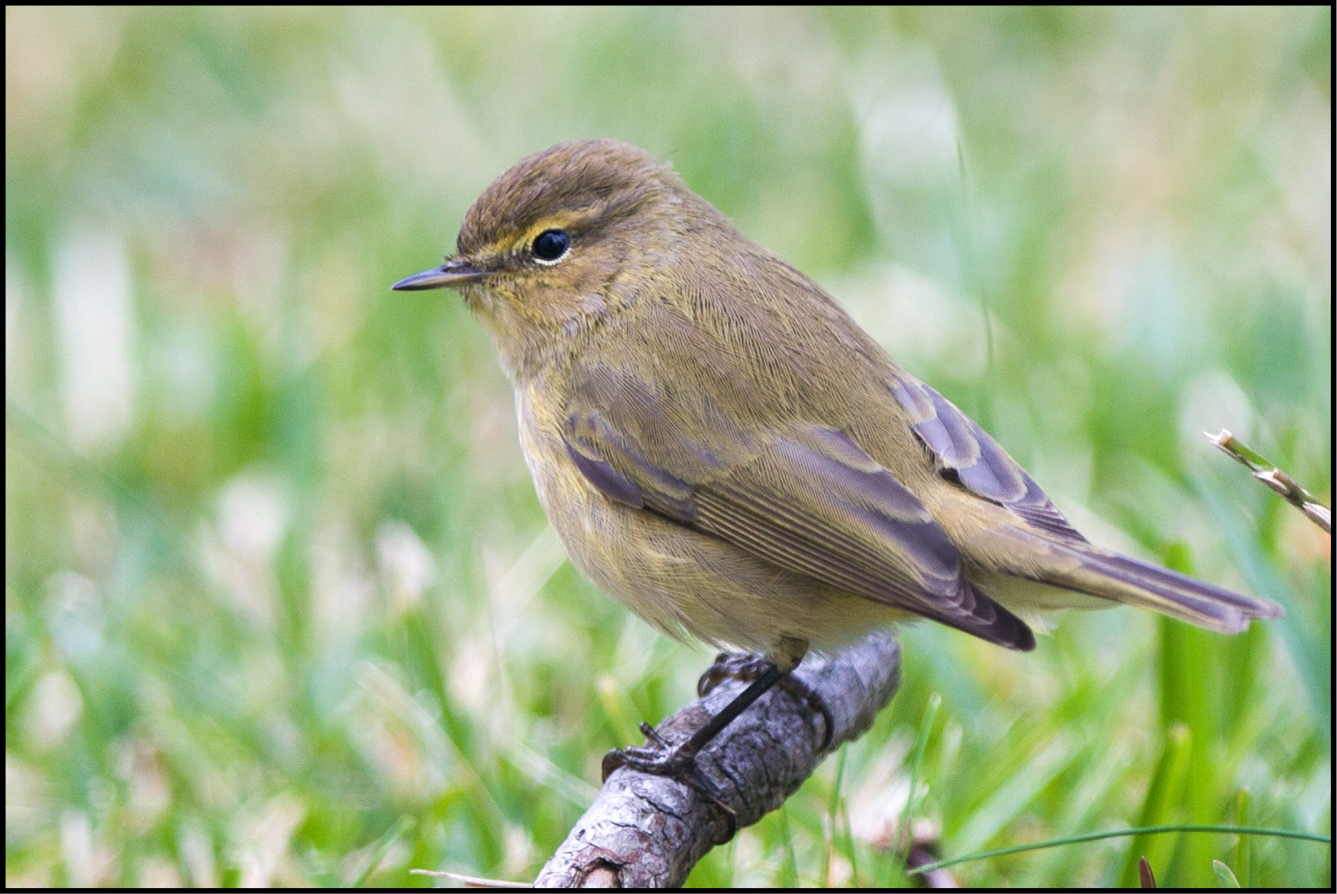
Photograph of Common Chiffchaff nominate subspecies from wintering grounds in Portugal (*Sergei Drovetski*).

The Common Chiffchaff (Fig 1) is divided into six subspecies [3]: *P*. *c*. *collybita*, breeding in western and central Europe east through Poland and Romania, the longer winged *P*. *c*. *abietinus* breeding in northern and eastern Europe, *P*. *c*. *brevirostris* breeding in western and northern Anatolia, *P*. *c*. *caucasicus* breeding in the Greater and Lesser Caucasus, *P*. *c*. *menzbieri*, believed to be restricted to the mountains of northeastern Iran and Turkmenistan, and *P*. *c*. *tristis* (‘Siberian Chiffchaff’) breeding from Arkhangelsk and the Pechora basin to the eastern limits of the species range [4,5]. Although the mean biometric and plumage differences between most of the subspecies are very subtle (summarized in [6]), most of the six subspecies are widely recognized [6]. The status of *P*. *c*. *brevirostris* is poorly resolved–it is not readily diagnosable by plumage from *P*. *c*. *abietinus* and some authors treat it as a synonym [2,7]. *P*. *c*. *caucasicus* was only described in 1991 [8], but any plumage differences from *P*. *c*. *brevirostris* remain undefined. All these subspecies of the Common Chiffchaffs might represent incipient or cryptic species.

Birds inhabiting some stretches of the Common Chiffchaff range still await taxonomic assignment. For example, chiffchaffs from southernmost Armenia (south of Goris), and northern Iran, between the ranges of *P*. *c*. *menzbieri* and *P*. *c*. *caucasicus*, have not been assigned to subspecies [1,4]. In the original description of *P*. *c*. *caucasicus* [8], Loskot discussed phenotypic differences between the birds from the Caucasus and Elburs mountains (northern Iran) but did not assign the latter distribution to a particular subspecies.

Previously, all chiffchaff taxa were included in a single species, *P*. *collybita* s.l. [7], though the lack of interbreeding and plumage differences between *P*. *c*. *caucasicus* and *P*. *sindianus lorenzii* in partial sympatry in the Caucasus led to them being split [9]. In the first molecular study of the complex [10], the authors used partial sequences of the mtDNA cytochrome-*b* gene (300–1041 base pairs (bp)) and found significant divergence among multiple lineages. Joint evaluation of mtDNA, acoustic, and phenotypic differences led to splitting of Iberian Chiffchaff and Canary Islands Chiffchaff, from Common Chiffchaff and confirmed species status of Mountain Chiffchaff [6]. The mtDNA of the former was the most distinct of the four species but the relationships among mtDNA lineages of the three latter species were unresolved [10]. That study also showed that the Common Chiffchaff was composed of five mtDNA clades that appeared to correspond to named subspecies (nominate *collybita*, *abietinus*, *tristis*, *brevirostris* and *caucasicus*), but the single deposited sequences of *brevirostris* and *caucasicus* were only 2 bp different (0.2% uncorrected), casting doubt on the genetic distinctiveness of these taxa as all other subspecies of *P*. *collybita* were 1.0–1.9% divergent. No samples of *P*. *c*. *menzbieri* were included in that study, but a single unpublished sequence (GenBank accession AF136374) confirms that this subspecies is also 1–2% divergent from other populations of *P*. *collybita*.

The correspondence between mtDNA structure and subspecific taxonomy of the Common Chiffchaff could be an artifact of the sampling employed. In particular, the authors sampled small numbers of each taxon from a single geographic area, except *P*. *c*. *collybita* (2 areas), and these areas were far away from contact zones between neighboring subspecies (Table 1 in [10]). mtDNA introgression has subsequently been discovered in two contact zones: between *P*. *c*. *abietinus* and *P*. *c*. *collybita* in Sweden [11] and between *P*. *c*. *abietinus* and *P*. *c*. *tristis* in the southern Urals and Arkhangelsk region [5,12,13]. There are still many unresolved questions about the mitochondrial and nuclear genetic distinctiveness of these and other chiffchaff taxa that can only be answered by deeper genetic analysis of many more individuals from across their ranges.

In order to evaluate geographic and taxonomic structure, we explored sequence variation of the mtDNA ND2 gene and a nuclear sex specific marker—intron 9 of the Z-linked aconitase 1 gene (ACO1I9) of the chiffchaff species complex. We used a Z-linked marker rather than an autosomal marker because the former has a smaller effective population size (N_e_) and thus its lineage sorting is faster than in the latter. Compared to earlier work [10], we substantially increased sample size, expanded geographic breadth of sampling within the Common Chiffchaff range, and included two populations outside of the range with unknown taxonomic status: from Transcaucasia (southernmost Armenia) and a breeding population recently discovered by us on Mt. Hermon (Anti-Lebanon Mountains).

## Material and methods

**Fig 2:**
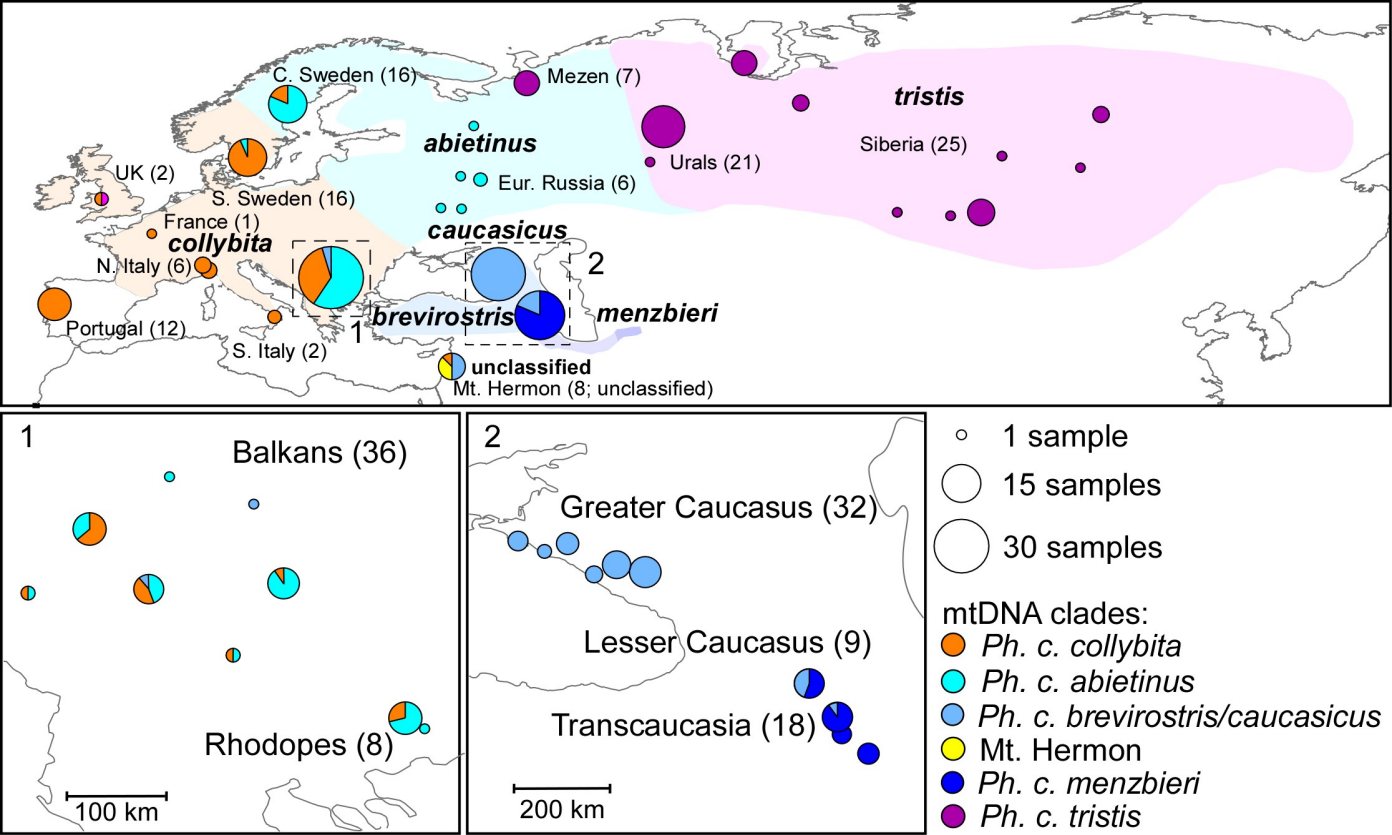
Ranges of Common Chiffchaff subspecies (different colors), sample localities and mtDNA clades. (Top). Sampling localities of Common Chiffchaffs for this study are represented by filled circles of size proportionate to the number of individuals. The common accepted ranges of the subspecies of Common Chiffchaff are shaded and labeled. Mitochondrial ND2 haplotypes of Common Chiffchaff were represented by six well separated clades, either novel (yellow) or assignable to a subspecies or subspecies group, shaded different colors in proportion to the number of birds in each clade at each sampling location. See text for justification of assigning ND2 clades to subspecies. Two small squares under the large map show respective expanded areas of the squares with dashed borders and identified by numbers (1 and 2). In southeast Europe (1), *P*. *c*. *abietinus* alleles predominate in the east and *P*. *c*. *collybita* in the west, though birds with *P*. *c*. *brevirostris/caucasicus* alleles were also found. In the Caucasus and Transcaucasia (2) there is a potential zone of introgression between *P*. *c*. *brevirostris/caucasicus* and *P*. *c*. *menzbieri*.

This study did not require ethical approval in our institutions because we used samples loaned to us by public museums or universities (S1 File) who comply with relevant regulations for acquisition and curation of their collections. We used 230 blood or tissue samples of the Common Chiffchaff from across its range (Fig 2 and S1 File). Our samples mostly comprised of local breeding or juvenile birds, with the exception of 14 wintering birds (12 from Portugal, 1 from Israel, and 1 from the United Kingdom). We also used five Iberian Chiffchaffs (breeding), 13 Mountain Chiffchaffs (six *P*. *sindianus sindianus* and seven *P*. *s*. *lorenzii*, 12 breeding, 1 hatchling) and seven Canary Islands Chiffchaffs sampled on February 26–28 on La Palma Island. The breeding season of the Canary Islands Chiffchaff runs from January through June [14], suggesting that our samples represent the breeding population of La Palma Island despite the early sampling dates. The end of February is within the winter/migration period of the common and Iberian Chiffchaffs in the Canary Islands, but occurrence of both species appears to be restricted to easternmost islands of the archipelago and neither species is known to occur on western Canary Islands including La Palma [15]. We used three Willow Warblers *Phylloscopus trochilus* as the closest known outgroup of the chiffchaff species complex [16].

Total genomic DNA extraction, mtDNA ND2 gene (1041 bp) amplification and sequencing followed the protocol described in detail [17]. Cytochrome b was sequenced for some individuals following the protocol of [10], to confirm that our assignment of ND2 haplotypes to Common Chiffchaff subspecies was consistent with that paper. GenBank accession numbers for these sequences are given in S1 File. We did not sample birds known to be relatives and all sequences were checked for pseudogenes and nuclear ND2 copies [18]. We also sequenced intron 9 of the Z-linked aconitase 1 gene (ACO1I9; 989 bp) using the PCR protocol and primers describes by Drovetski et al. [19].

Sequences were aligned automatically in Sequencher 5.0.1 (Gene Codes Corporation, Ann Arbor, MI) and verified manually to ensure consistent alignment of two indels in the ACO1I9 sequences: insertion at sites 123–127 in four individuals from southern Armenia and a deletion at sites 956–959 in a single individual from the northern Urals. ACO1I9 alleles of heterogametic males with multiple nucleotide differences were resolved using PHASE 2.1.1 [20]. We conducted two independent runs in which the first 500 iterations were discarded as burn-in. The following 5000 iterations used a thinning interval of 10. Known haplotypes from females, homozygous males, and males with a single polymorphic site were set as known alleles.

The Bayesian Information Criterion implemented in JModelTest 2.1.1 [21] was used to select the best-fit model (HKY+G) of sequence evolution for ND2: Lset base = (0.3039 0.3474 0.1018) nst = 6 rmat = (7.2523 228.8968 7.1228 1.0000 132.6996) rates = equal pinvar = 0.6640. A time-calibrated mtDNA gene tree was reconstructed in BEAST 2.4.2 [22] using the mean rate of sequence evolution (2.9 x 10^−2^ substitutions/site/Ma) calculated for the ND2 gene from Hawaiian Honeycreepers *Drepanidinae* [23]. This calibration is probably the most robust available for passerines. We selected the Yule process speciation prior and conducted two independent runs with strict and relaxed lognormal clock priors. Yule tree prior assumes a constant lineage birth rate for each branch in the tree. This tree prior is the most suitable for trees describing the relationships among individuals from different species. Since the maximum likelihood ratio test [24] showed no significant differences between tree likelihoods (ΔL = 122.938, df = 251, P = 1) of clock like and non-clock (log normal) trees, we present the tree based on the strict clock. We conducted two independent runs with chain length of 10^8^ generations sampled every 10^4^ generations. The first 10^4^ trees from each run were discarded as a burn-in. Tracer 1.6 (http://beast.bio.ed.ac.uk/Tracer) was used to determine the effective sample size and calculate the mean and 95% highest posterior density interval (95% HPD) for each parameter. We used LogCombiner 2.4.2 [25] to combine results of the two independent runs. Tree topology was assessed using TreeAnnotator 2.4.2 [25] and visualized in FigTree 1.4.1 (http://tree.bio.ed.ac.uk/software/figtree/).

For chiffchaff populations we estimated nucleotide diversity [26], haplotype diversity [26], theta per site from the number of polymorphic sites per nucleotide [27] and Tajima’s D [28] using DnaSP software v5.10 [29] for both ND2 and ACO1I9 genes. We used TCS v.1.21 [30] for constructing a ACO1I9 haplotype network. Indels were treated as missing data. We used AMOVA implemented in Arlequin 3.5.2.2 [31] to asses inter-regional differentiation in ACO1I9 allele frequencies and divergence. Level of divergence at ACO1I9 was low and in order to present the unbiased dataset, allele divergence was measured as uncorrected *p*-distance and sites with indels were excluded only if they were present in pairwise comparisons. We used the Principal Component Analysis (PCA) implemented in Genalex (http://biology-assets.anu.edu.au/GenAlEx/Welcome.html) to summarize the matrix of pairwise Φ_st_ values based on ACO1I9 sequences and hence estimate the proportion of genetic diversity attributable to differences among populations.

## Results

### mtDNA phylogeography

**Fig 3:**
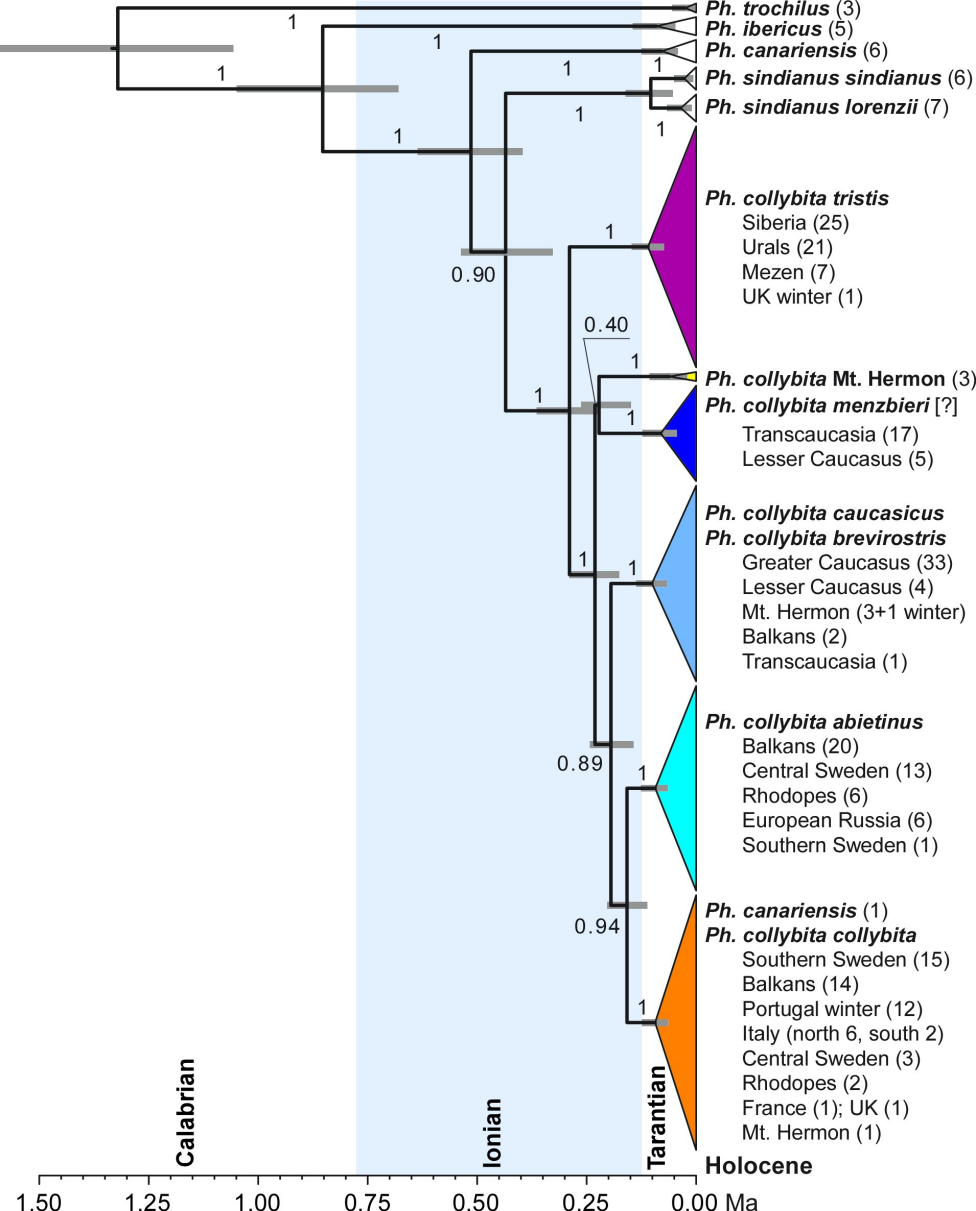
Dated phylogenetic relationships of mtDNA ND2 haplotypes. Time calibrated Bayesian tree representing relationships among ND2 clades of Common Chiffchaffs, other chiffchaff species and Willow Warbler. Localities, inferred subspecies assignment, and numbers of birds sampled are listed on the right. Numbers next to branches indicate their posterior probability values. Gray bars next to nodes represent 95% HPD intervals for their age estimates. The chiffchaff complex was estimated to be evolutionarily young. The divergence of all chiffchaff species and clades within the Common Chiffchaff was estimated to have happened during the Ionian stage (middle Pleistocene). Two clades of the Mountain Chiffchaff were estimated to have diverged in the Tarantian stage (late Pleistocene).

Common Chiffchaffs yielded six distinct clades of mitochondrial ND2 sequences. The geographical distribution of birds falling within these clades is shown in Fig 2. The monophyly of the chiffchaff species complex as a whole was strongly supported (PP = 1) relative to the outgroup (Willow Warbler) in our mtDNA ND2 gene tree (Fig 3). The ND2 tree topology recovered as monophyletic (all PP = 1) the four currently recognized species, with the exception of a single bird from La Palma Island (Canary Islands) that was included into one of the Common Chiffchaff clades (west European, see below). The Iberian Chiffchaff and Canary Islands Chiffchaff were each represented by a single clade. The Mountain Chiffchaff consisted of two sister clades (PP = 1) corresponding to its subspecies *P*. *s*. *sindianus* and *P*. *s*. *lorenzii* (both PP = 1). The Common Chiffchaff was monophyletic (PP = 1) and its six subclades were strongly supported (PP = 1).

Mitochondrial DNA was used to estimate of divergence time of chiffchaff lineages [32–34]. The most divergent among the chiffchaff complex clades was the Iberian Chiffchaff (PP = 1) which we estimated (taking assumed generation time as 1 year) to have diverged from the common ancestor of all other chiffchaffs (PP = 1) at the end of the Calabrian stage of the Pleistocene, approximately 0.853 Ma (95% HPD interval 0.680–1.050 Ma; Fig 3). The common ancestor of the Canary Islands Chiffchaff diverged from the ancestor of the Mountain and Common Chiffchaffs during the Ionian stage of the Pleistocene approximately 0.514 Ma (0.369–0.637 Ma). Monophyly of the Mountain and Common Chiffchaffs, however, was only marginally supported (PP = 0.90). Therefore, we cannot reject a trichotomy comprising these two species and the Canary Islands Chiffchaff. The Mountain and Common Chiffchaffs diverged during the Ionian stage of the Pleistocene approximately 0.435 Ma (0.327–0.537 Ma).

Two Mountain Chiffchaff subspecies appear to have diverged during the Tarantian stage of the Pleistocene approximately 0.104 Ma (0.053–0.161 Ma). Each of these two clades was represented by a single haplotype shared by all individuals of *P*. *s*. *sindianus* (n = 5) or *P*. *s*. *lorenzii* (n = 7), respectively. The transition from the Calabrian to Ionian stage was characterized by the switch from 41 Ka low-amplitude glacial cycles to 100 Ka cycles of much greater amplitude and severity that had profound effect on terrestrial biota [35]. Ionian high-amplitude glaciation cycles may have resulted in geographic structuring of mtDNA lineages in the chiffchaff species complex.

The western Common Chiffchaff clades were geographically structured and substantially corresponded with the known ranges of currently accepted subspecies. The diversification within the Common Chiffchaff began with the separation of the eastern clade (Siberia, Urals, and Mezen, corresponding to ‘Siberian Chiffchaff’ *P*. *c*. *tristis*) from the common ancestor of the five other Common Chiffchaff clades in the Ionian stage of the Pleistocene approximately 0.289 Ma (0.220–0.365 Ma). Although the monophyly of each of the five western clades was strongly supported (PP = 1), the relationships among them were unresolved (0.40 < PP < 0.94; Fig 3). The ages of these divergence events ranged from 0.158 (0.111–0.234 Ma) to 0.231 Ma (0.175–0.290 Ma).

Each of the five western clades dominated a different part of the western Palearctic but multiple clades were detected in a number of regions. The two most recently diverged clades occupied Europe (Figs 2 and 3). The ‘east European’ clade was found in European Russia south of 61^o^N and west of 44^o^E (6 birds) and also dominated central Sweden (13 of 16 birds), Rhodope Mountains (six of eight birds), and the Balkans (20 of 37 birds). It can confidently be assigned therefore to *P*. *c*. *abietinus*. One of 16 birds in southern Sweden also carried an east European *abietinus* haplotype.

Western-most Europe was dominated by a clade that was found in all 12 birds wintering in Portugal, single birds sampled in the UK and France, all 8 Italian birds, and 15 of 16 birds sampled in southern Sweden (Figs 2 and 3). This clade corresponds geographically with nominate *P*. *c*. *collybita*. This clade was also comprised of 14 of 37 birds from the Balkans (most common in the western Balkans–see Fig 2), in two of eight birds from Rhodope Mountains (Greece), three of 16 birds from central Sweden, one of 8 birds from Mt. Hermon, and 1 out of 7 birds from La Palma Island (Canary Islands; Figs 2 and 3).

A third western clade (southern) was the only clade among Common Chiffchaffs sampled from the Greater Caucasus (n = 33; Figs 2 and 3). This clade was also comprised by four of nine birds from the Lesser Caucasus, one of 18 birds from Transcaucasia, four of eight birds from Mt. Hermon, and two of 37 birds from the Balkans. Birds breeding in the Greater Caucasus are, by definition, *P*. *c*. *caucasicus*; however this subspecies is not genetically distinct from *P*. *c*. *brevirostris* of Turkey based on *cytb* ([10]; see above). The fact that individuals with this ‘*caucasicus*’ ND2 sequence were found at such widely spaced localities as the Balkans and Mt. Hermon, much closer to the known range of *brevirostris* is consistent with the assignation of this third Common Chiffchaff clade to a combined ‘*P*. *c*. *brevirostris/caucasicus*’ (Figs 2 and 3). The 1041 bp *cytb* sequences of the four Mt. Hermon birds in this clade was also obtained (S2 File): these were identical and only 2–3 bp different from the *P*. *c*. *brevirostris* and *caucasicus* sequenced by [10], thus we are confident of the assignation of this southern clade to represent combined *P*. *c*. *brevirostris/caucasicus* (Fig 3).

The most divergent among western chiffchaffs were two sister clades occupying southeastern limits of the species range. The first clade was dominated by birds from Transcaucasia (17 of 18 birds) and the Lesser Caucasus (five of nine birds), and is most likely to represent *P*. *c*. *menzbieri*. The other was represented by just three of eight birds sampled on Mt. Hermon (Anti-Lebanon) and this clade represents a novel mitochondrial haplotype not assignable to any known subspecies. The *cytb* sequences of these birds also confirmed this conclusion, being highly divergent from any sequences identified by [10].

The pairwise divergences between ND2 sequences of chiffchaff taxa are summarized in Table 1. Genetic diversity indices within populations of mtND2 gene were generally very similar among populations (Table 2). Haplotype diversities were lowest in South Sweden and in Transcaucasia (0.542 and 0.556 respectively) and highest in Rhodopes and Urals (1 and 0.981 respectively). Nucleotide diversity varied from 0.00144 in the South Sweden and Italy to 0.01107 at Mount Hermon. In general, nucleotide diversity was highest in mixed populations, although South Sweden had the lowest diversity (Table 2). For most populations Tajima’s D values were negative, but not significant.

**Table 1:** Mean pairwise sequence divergence (uncorrected *p*-distance) among ND2 clades (Fig 3) of the chiffchaff complex and Willow Warbler *P*. *trochilus*.

	1	2	3	4	5	6	7	8	9	10
1. *Ph*. *trochilus*										
2. *Ph*. *ibericus*	0.10112									
3. *Ph*. *canariensis*	0.09206	0.06571								
4. *Ph*. *s*. *sindianus*	0.09286	0.06359	0.04195							
5. *Ph*. *s*. *lorenzii*	0.09094	0.06167	0.03810	0.00576						
6. *Ph*. *c*. *tristis*	0.08590	0.06017	0.03823	0.03198	0.03102					
7. *Ph*. *c*. *novel_lineage*	0.09638	0.06462	0.03971	0.03330	0.03234	0.01824				
8. *Ph*. *c*. *menzbieri*	0.09290	0.06103	0.03736	0.03096	0.03000	0.01694	0.01448			
9. *Ph*. *c*. *caucasicus*	0.09467	0.06150	0.03710	0.03257	0.03161	0.01857	0.01601	0.01384		
10. *Ph*. *c*. *abietinus*	0.09328	0.05782	0.03198	0.02759	0.02663	0.01538	0.01283	0.01051	0.01025	
11. *Ph*. *c*. *collybita*	0.09023	0.05789	0.03481	0.02649	0.02557	0.01624	0.01374	0.01126	0.01115	0.00615

**Table 2:** Population genetic diversity values estimated from the mtND2 sequences (1041 bp) of all sampled populations of *Phylloscopus collybita* except wintering population in Portugal.

Population	N of individuals	N of haplotypes	Haplotype diversity	Nucleotide diversity	Theta per site (S)	Tajima's D test
Siberia	25	18	0.927	0.00217	0.00611	**-2.36871**
Urals	21	18	0.981	0.00309	0.00641	**-1.97437**
Mezen	7	6	0.952	0.00311	0.00353	-0.62991
**Mount Hermon**	7	5	0.905	0.01107	0.00902	1.28547
**Transcaucasia**	18	5	0.556	0.0021	0.00475	**-2.24653**
**Lesser Caucasus**	10	7	0.911	0.00826	0.00679	1.01752
Greater Caucasus	32	12	0.861	0.0017	0.0031	-1.47367
European Russia	6	5	0.933	0.00224	0.00294	-1.39031
**Central Sweden**	16	10	0.867	0.00275	0.00405	-1.25424
**Rhodopes**	8	8	1	0.00374	0.00445	-0.79932
**Balkans**	36	20	0.906	0.00437	0.00741	-1.443
**South Sweden**	16	6	0.542	0.00144	0.00347	-2.23554
Italy	8	4	0.643	0.00144	0.00222	-1.63982

Significant values of Tajima's D test in are highlighted in bold. Populations highlighted in bold indicate mixed populations where at least two lineages occur sympatrically.

### ACO1I9 variation

**Fig 4:**
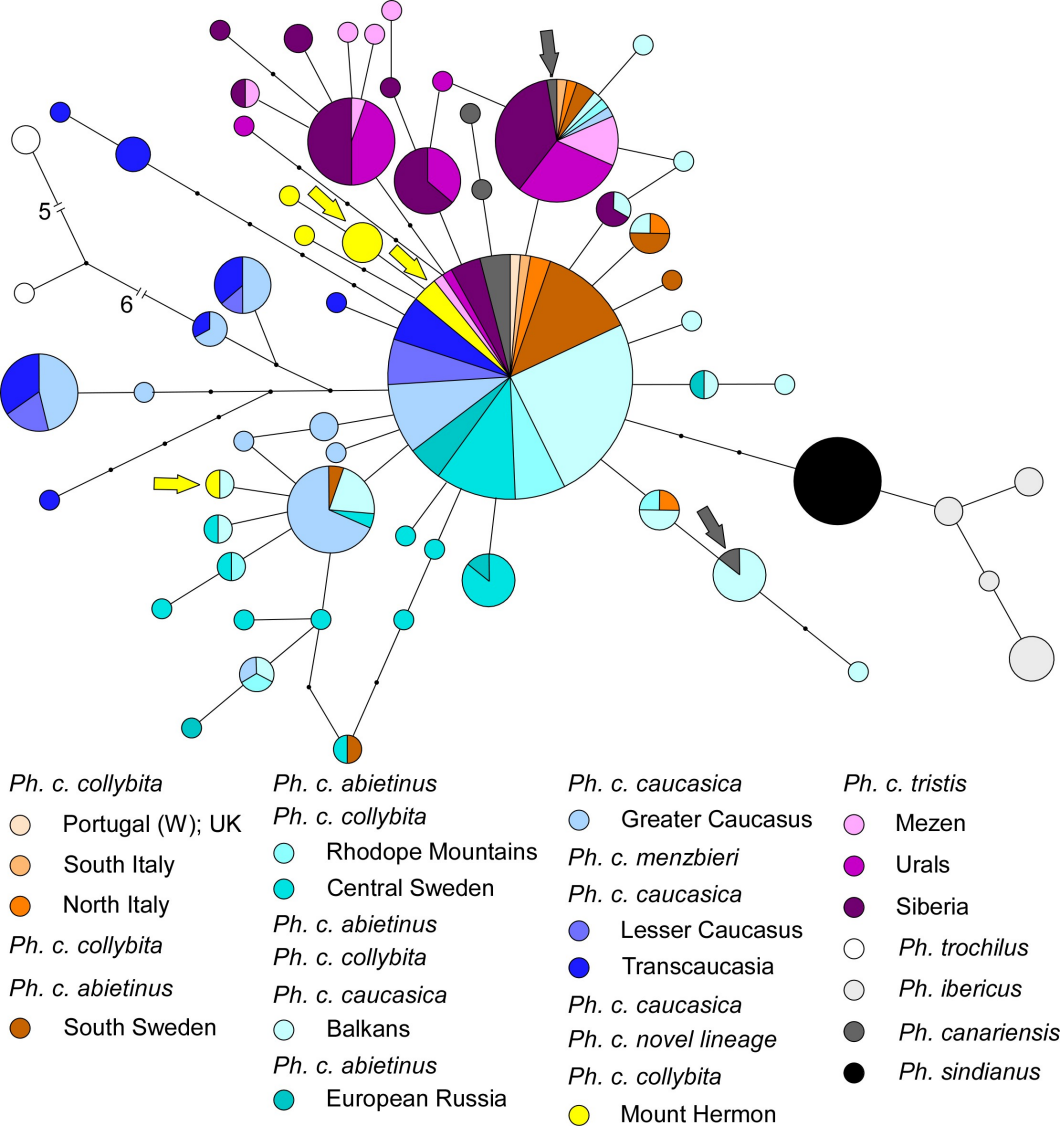
Network of ACO1I9 alleles. Each circle represents an ACO1I9 allele. Circle area is proportional to the number of individual sequences obtained that corresponded to that allele. Colors represent sampling location (not necessarily ND2 clade assignment). In the localities with mtDNA clades representing multiple subspecies, the dominant one is listed first. Sectors within circles are sized proportionate to number of sequences obtained from birds at each sampling location with that allele. Grey arrows represent the ACO1I9 alleles of the bird sampled at La Palma (Canary Islands) that had a *P*. *c*. *collybita* ND2 haplotype. Yellow arrows indicate ACO1I9 alleles obtained from Mt. Hermon birds with the novel unassigned ND2 haplotype. Allele sharing between birds of different species or subspecies may be due to introgression or retention of ancestral shared sequences.

In contrast to mtDNA ND2 haplotypes, lineage sorting among ACO1I9 alleles was incomplete across the four species complex as a whole. Population genetic diversity is summarized in Table 3. The Iberian and Mountain Chiffchaffs had unique ACO1I9 alleles not found in other species (Fig 4). Lineage sharing was observed between the Canary Islands Chiffchaff and Common Chiffchaff (Fig 4). Only two of 10 Canary Islands Chiffchaff sequences represented unique haplotypes. Six other ACO1I9 alleles from la Palma Island birds with *canariensis* ND2 clade were identical to the predominant Common Chiffchaff allele (shared by 148 Common Chiffchaffs representing all sampling locations). The male individual from the Canary Islands that had ND2 characteristic of nominate *P*. *collybita* was heterozygous at ACO1I9, carrying one allele identical with the second most predominant Common Chiffchaff allele (37 birds from across Europe and Russia) and one allele found in six Common Chiffchaffs from the Balkans (Fig 4)–alleles not observed in any bird with a *canariensis* ND2 haplotype.

**Table 3:** Population genetic diversity values estimated from the ACO1I9 sequences (989 bp) of all sampled populations for local breeding individuals only.

Population	N of individuals	N of sampled alleles	N of haplotypes	Haplotype diversity	Nucleotide diversity	Theta per site (S)	Tajima's D test
Siberia	22	38	8	0.788	0.00192	0.00193	-0.00978
Urals	21	34	8	0.795	0.00199	0.00174	0.41403
Mezen	5	9	4	0.694	0.00169	0.00223	-1.06907
Mount Hermon	7	12	6	0.758	0.00138	0.00201	-1.19623
Transcaucasia	17	25	8	0.737	0.0026	0.00375	-1.06813
Lesser Caucasus	8	13	3	0.5	0.00156	0.00163	-0.16103
Greater Caucasus	31	52	15	0.85	0.00254	0.00313	-0.57249
European Russia	6	10	4	0.533	0.00117	0.00179	-1.38818
Central Sweden	16	32	12	0.728	0.00153	0.00251	-1.22364
Rhodopes	7	14	5	0.505	0.00099	0.00191	-1.72892
Balkans	35	63	16	0.646	0.00125	0.003	-1.70491
South Sweden	13	26	6	0.468	0.00076	0.00185	**-1.80814**
Italy	7	10	4	0.644	0.00076	0.00107	-1.03446

Significant values of Tajima's D test are highlighted in bold.

All Mountain Chiffchaffs (*P*. *s*. *sindianus* and *P*. *s*. *lorenzii*) shared a single unique allele, so in contrast to mtDNA, ACO1I9 failed to distinguish these subspecies (Fig 4).

All subspecies of Common Chiffchaff shared ACOI9 sequences with all other subspecies. Most birds from Siberia, the Urals, and Mezen, all of which were included in the most divergent northeastern *P*. *c*. *tristis* mtDNA clade, had ACO1I9 alleles unique to that clade. However other birds from these areas (all with *P*. *c*. *tristis* ND2) shared the two most common alleles with birds from elsewhere. The proportion of sequences from Siberia/Urals/Mezen among those with the most common allele was low—11 of 154, whereas there were 30 sequences from Siberia/Urals/Mezen of the total of 38 sequences recovered as the second most common allele.

The three birds from Mount Hermon with novel unclassified ND2 sequences also shared some ACO1I9 alleles with Common Chiffchaffs from elsewhere. One male bird was homozygous for a private allele (Fig 4). Another male was heterozygous, carrying this novel allele and the predominant allele shared with all other localities. The third bird, a female, had a single ACO1I9 allele shared with a single bird from the Balkans (Fig 4). Two other unique alleles were found in Mt. Hermon birds with *P*. *c*. *brevirostris/caucasicus* ND2, but all others were the predominant allele.

Our AMOVA revealed significant differentiation between eastern (Siberia/Urals/Mezen) and all others localities that explained 15.4% of the total variance in the Common Chiffchaff dataset (P = 0.005 ± 0.002). Differences among localities within regions accounted for 6.9% of the variance (P < 0.001) and differences among individuals within localities accounted for 77.7% of the variance (P < 0.001). Pairwise comparisons also showed strong differentiation of each eastern locality from all western localities (Φ_st_ = 0.066–0.220, P = 0–0.5; Table 4) except for Italy vs. Mezen (Φ_st_ = 0.066, P = 0.11) and Italy vs. Urals (Φ_st_ = 0.110, P = 0.06) where differentiation was marginally significant. However, the three eastern localities (Siberia, Urals, Mezen) were not differentiated from each other (Φ_st_ = -0.019–0.024, P = 0.19–0.91; Table 4) indicating that differentiation among localities in our AMOVA was restricted only to the western part of the Common Chiffchaff range. Within the western regions, there was no differentiation among European Russia, Rhodopes, Balkans, central or southern Sweden, and Italy (Φ_st_ = -0.028–0.051, P = 0.06–0.76; Table 4), except for the most geographically distant pair: central Sweden and Balkans (Φ_st_ = 0.059, P < 0.001; Table 4). Collectively, these localities were differentiated from the Greater Caucasus (Φ_st_ = 0.082–0.143, P = 0–0.07 ± 0.02), Lesser Caucasus (Φ_st_ = 0.111–0.151, P = 0–0.06 ± 0.02), and Transcaucasia (Φ_st_ = 0.099–0.199, P = 0–0.03 ± 0.02). The Lesser Caucasus was not differentiated from the Greater Caucasus (Φ_st_ = -0.006, P = 0.42) or Transcaucasia (Φ_st_ = -0.003, P = 0.41), although the latter two were differentiated from each other (Φ_st_ = 0.053, P = 0.01; Table 4). Only Mt. Hermon birds were differentiated from all other western localities in their ACO1I9 allele frequencies (Φ_st_ = 0.109–0.173, P = 0–0.02 ± 0.01; Table 4).

**Table 4:** Φ_st_ values based on ACO1I9 sequences (below the diagonal; negative numbers were replaced with 0) and their P-values (above diagonal) for pairwise comparisons of Common Chiffchaff regional samples.

	Siberia	Urals	Mezen	Mt. Hermon	Transcaucasia	Les. Caucasus	Gr. Caucasus	Eur. Russia	C. Sweden	Rhodopes	Balkans	S. Sweden	Italy
**Siberia**	*	**0.91±0.03**	**0.19±0.05**	**0**	**0**	**0**	**0**	**0**	**0**	**0.01±0.01**	**0**	**0**	**0.02±0.01**
**Urals**	**-0.019**	*	**0.19±0.04**	**0**	**0**	**0**	**0**	**0**	**0**	**0**	**0**	**0**	**0.06±0.02**
**Mezen**	**0.019**	**0.024**	*	**0.001±0.001**	**0**	**0.01±0.01**	**0**	**0**	**0**	**0**	**0**	**0**	**0.11±0.03**
**Mt. Hermon**	**0.216**	**0.211**	**0.225**	*	**0.01±0.01**	**0**	**0**	**0.01±0.01**	**0**	**0.01±0.01**	**0**	**0**	**0.02±0.01**
**Transcaucasia**	**0.225**	**0.219**	**0.152**	**0.139**	*	**0.41±0.05**	**0.01±0.01**	**0.02±0.01**	**0**	**0**	**0**	**0**	**0.03±0.02**
**Les. Caucasus**	**0.216**	**0.211**	**0.214**	**0.173**	**-0.003**	*	**0.42±0.04**	**0.05±0.02**	**0**	**0.03±0.02**	**0**	**0.01±0.01**	**0.06±0.02**
**Gr. Caucasus**	**0.228**	**0.229**	**0.184**	**0.144**	**0.053**	**-0.006**	*	**0.07±0.02**	**0**	**0.03±0.01**	**0**	**0**	**0.01±0.01**
**Eur. Russia**	**0.167**	**0.164**	**0.178**	**0.109**	**0.099**	**0.111**	**0.085**	*	**0.76±0.04**	**0.63±0.04**	**0.13±0.06**	**0.18±0.03**	**0.21±0.03**
**C. Sweden**	**0.198**	**0.201**	**0.18**	**0.12**	**0.167**	**0.138**	**0.112**	**-0.028**	*	**0.31±0.05**	**0**	**0.07±0.02**	**0.09±0.01**
**Rhodopes**	**0.151**	**0.154**	**0.153**	**0.109**	**0.115**	**0.118**	**0.082**	**-0.021**	**0**	*	**0.59±0.04**	**0.73±0.04**	**0.59±0.04**
**Balkans**	**0.203**	**0.212**	**0.186**	**0.127**	**0.199**	**0.141**	**0.143**	**0.025**	**0.059**	**-0.01**	*	**0.06±0.02**	**0.45±0.05**
**S. Sweden**	**0.175**	**0.183**	**0.195**	**0.147**	**0.156**	**0.151**	**0.12**	**0.024**	**0.031**	**-0.018**	**0.019**	*	**0.71±0.03**
**Italy**	**0.103**	**0.11**	**0.066**	**0.134**	**0.099**	**0.122**	**0.105**	**0.044**	**0.051**	**-0.015**	**-0.004**	**-0.02**	*

**Fig 5:**
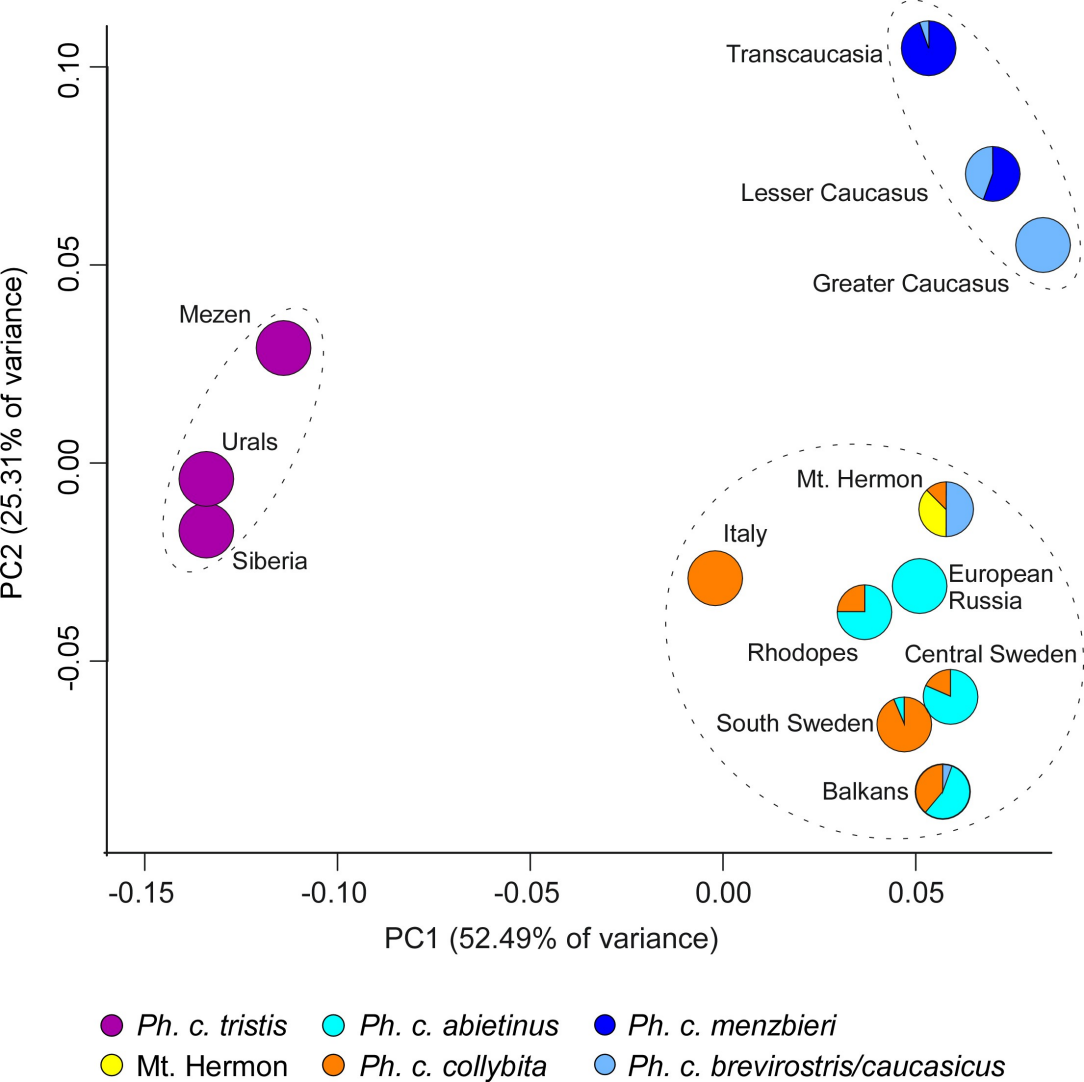
Plot of the PCA based on pairwise Φ_st_ values calculated using ACO1I9 data. Different colors represent proportions of different mtDNA clades sampled in each locality.

To visualize genetic differences among localities, we also performed a Principal Component Analysis of the pairwise Φ_st_ values calculated using ACO1I9 data. This PCA identified three distinct clusters of localities (Fig 5) representing three subspecies groups. The first principal component (PC1) explained 52.5% of the variance, and separated the three eastern localities in which all sampled birds carried *P*. *c*. *tristis* mtDNA as the most distinct among the three clusters. The second principal component (PC2) explained 25.3% of the variance, and separated the three southeastern localities (the Greater Caucasus, Lesser Caucasus, and Transcaucasia) from the other two clusters. This southeastern cluster was closer to the third cluster composed of East and West European localities and Mt. Hermon. The Lesser Caucasus, where birds carried almost equal proportion of Greater Caucasus and Transcaucasian mtDNA haplotypes was situated between those localities. Finally, in the European cluster, localities with mix of East and West European mtDNA haplotypes were closer to European Russia (that had birds with only East European haplotypes) than to Italy (that had only West European Haplotypes).

## Discussion

### Patterns of genetic variation and biogeography in chiffchaffs

The landmark paper [10] which substantially underlies our understanding of the taxonomy of the chiffchaff complex, was based on analysis of very few individuals. Since then, chiffchaffs have remained patchily sampled geographically, with work focusing on status of the Iberian Chiffchaff *P*. *ibericus*, and on the intergrade zones between *P*. *c*. *abietinus* and *P*. *c*. *tristis* in Russia, or between *P*. *c*. *abietinus* and *P*. *c*. *collybita* in Sweden [5,11,12,36]. Very few chiffchaff sequences have been deposited in public databases. The current study samples many more individuals including those from neglected areas of potential intergradation such as the Balkans, the Caucasus and Transcaucasia, and describes for the first time the mixed genetic status of a previously unreported isolated breeding population on Mt. Hermon.

A combination of mitochondrial and Z-linked markers was used. The topology of our ND2 tree (Fig 3) was identical to that of the Neighbor-Joining tree of Helbig et al. [10] (their Fig 2, left) based on partial *CytB* sequence, but had stronger statistical support. Lineage sorting between the four species, Common, Iberian, Mountain and Canary Islands Chiffchaff, was virtually complete for mtDNA (Fig 3). Both trees suggested that the Iberian Chiffchaff is the most divergent among the members of the chiffchaff species complex. This initial divergence was followed by near-trichotomous split of the Canary Islands Chiffchaff, Mountain Chiffchaff, and Common Chiffchaff. Sequential divergence of small peripheral populations from a common ancestor with a large range suggests a possible peripatric mode of speciation. Peripatric speciation has been shown to be a common geographic mode of speciation in other boreal and temperate avian taxa [37,38] and may have contributed to separation of chiffchaff lineages.

mtDNA variation was structured within both Mountain and Common Chiffchaffs. In the Mountain Chiffchaff, mtDNA sequences were divided into two clades each representing one of the two allopatric subspecies *P*. *s*. *sindianus* (Pamir and Tian-Shan) and *P*. *s*. *lorenzii* (Caucasus, eastern Turkey, northwestern Iran). The lack of variation within both clades was especially surprising in *P*. *s*. *lorenzii* because four birds were sampled in the western Greater Caucasus and three birds in the central part of the Lesser Caucasus (S1 File), approximately 465 km apart. It is possible that after the initial split of the two Mountain Chiffchaff subspecies, probably in glacial refugia [39], both experienced severe bottlenecks.

In contrast to the two Mountain Chiffchaff subspecies, none of the currently recognized Common Chiffchaff subspecies [4] are fully geographically isolated. However, distinct mitochondrial clades were evident corresponding with the established subspecies taxonomy:

*P*. *collybita collybita* (Western and Central Europe, into Sweden and Southeastern Europe and the Middle East);
*P*. *c*. *abietinus* (Eastern and Northern Europe, European Russia);
*P*. *c*. *tristis* (Far Northeastern Europe, east into Asia);
*P*. *c*. *brevirostris/caucasicus* (Balkans through to Greater and Lesser Caucasus, Transcaucasia and the Middle East);
*P*. *c*. *menzbieri* (Transcaucasia, east into Transcaspia)

In addition, a novel divergent haplotype was identified in three birds from Mount Hermon that was not assignable to any known subspecies.

The range of *P*. *c*. *tristis* was previously thought to extend from the Pechora basin eastwards to middle Kolyma [1,6,16,40]. However, the presence of only the eastern haplotypes in Mezen, 365 km west of the Pechora River, is consistent with recent data showing that *P*. *c*. *tristis* is distributed much further west in northern Europe than previously thought [5,13].

As may be expected of a nuclear gene in a young evolving complex, ACO1I9 variation was not as geographically and taxonomically structured as mtDNA ND2. There are three confounding factors that contribute to a more limited structure of Z-chromosome (sex) linked loci. First, the effective population size of the Z-linked loci is three-fold higher than that of mtDNA, resulting in three-fold longer time required for completion of lineage sorting. Second, the substitution rate of ACO1I9 is several-fold lower than the substitution rate of the mtDNA ND2 gene [19,23,41]. Finally, in contrast to mtDNA that is associated with females, often the more dispersive sex in birds [42], Z-linked loci are associated primarily (⅔ of Z-linked alleles) with potentially more philopatric males and therefore are more likely to introgress between populations in the secondary contact zones [43–45].

Despite the limited resolution of ACO1I9, Iberian, Common and Mountain Chiffchaffs shared no ACO1I9 alleles, suggesting they were genetically differentiated at both Z-linked and mitochondrial loci.

Unlike Mountain and Iberian Chiffchaff, some individuals of Canary Islands Chiffchaff, carrying *P*. *canariensis* ND2 alleles, shared ACO1I9 alleles with Common Chiffchaff. Indeed, eight of 10 ACO1I9 sequences we sampled were shared with the Common Chiffchaff. One single presumed *P*. *canariensis* individual from La Palma Island (Canary Islands) had an ND2 sequence that was included in the west European clade, diagnostic of nominate Common Chiffchaff, and alleles of ACO1I9 that were also consistent with Common Chiffchaff and not found in any other *P*. *canariensis* in our sample. Although nominate *collybita* is not known to occur on La Palma, silent birds would be difficult to identify, and it seems likely that this individual was a wintering individual of that subspecies. In spite of their different songs, we speculate that hybridization may occur between these species, at least occasionally.

Within the Common Chiffchaff, our AMOVA results for ACO1I9 were consistent with the mtDNA gene tree. The PCA of the pair-wise Φ_st_ values for Common Chiffchaffs (Fig 5) also supported strong similarity of the mtDNA phylogenetic signal and the degree of differentiation among geographic regions in ACO1I9 allele frequency and divergence. Similar to the mtDNA gene tree, eastern localities with *P*. *c*. *tristis* mtDNA formed a cluster that was most divergent from all other localities. Sequences from Transcaucasia, the Greater and Lesser Caucasus formed a cluster on the PCA chart (Fig 5), representing birds with ND2 of *P*. *c*. *brevirostris/caucasicus* and *menzbieri*, that was distant from the single cluster of European localities hosting two sister European mtDNA clades (*P*. *c*. *collybita* and *P*. *c*. *abietinus*).

The great deal of resolution of Common Chiffchaff subspecies at ND2 together with extensive lineage sharing between discernable subspecies groups at ACO1I9 is consistent with divergence between 1 and 3 N_e_ generations ago.

Our study did not have power to determine the extent to which lineage sharing at ACO1I9 between Canary Islands Chiffchaff and Common Chiffchaff, and between Common Chiffchaff subspecies, was due to retention of ancestral shared alleles or hybridization/introgression in zones of range overlap. However previous data and our ND2 analysis from multiple zones of overlap indicates that introgression does occur.

Although our data did not show widespread mtDNA introgression between the *tristis* clade and its neighbor the *abietinus* clade (Fig 2), a narrow introgression zone has been discovered in the southern Urals [12] and recently in the Arkhangelsk region [5]. A large proportion of males in the hybrid zone exhibit intermediate phenotypic characters and a mix of genetic ancestry, indicating ongoing and past gene flow [5].

#### The southern subspecies group

The ranges of three southern subspecies *P*. *c*. *brevirostris*, *P*. *c*. *caucasicus*, and *P*. *c*. *menzbieri* are poorly known and information about their contact zones is generally lacking [1,6,16], however the PCA clustered southern birds together on basis of ACO1I9. Available information suggests that *P*. *c*. *brevirostris* inhabits the western and northern Anatolian Peninsula (Turkey), *P*. *c*. *caucasicus* breeds in both Caucasus ranges and south to Goris in the Armenian Highlands, and that *P*. *c*. *menzbieri* is known from northeastern Iran and the Kopet Dag Mountains along the southern border of Turkmenistan. While we did not have samples from Turkey, the mtDNA clade that we found in all 32 birds from the breeding population of the Greater Caucasus (assignable definitively on location to *caucasicus*) and in four of nine birds from the Lesser Caucasus was also discovered in one bird from Transcaucasia, two birds from Balkans and four of eight birds from Mt. Hermon (Fig 2), suggesting a single genetic *P*. *c*. *brevirostris/caucasicus* clade that may introgress with nominate *P*. *c*. *collybita* and *P*. *c*. *abietinus* in the Balkans.

The central part of the Lesser Caucasus and Transcausasia south to Iranian border were dominated by a genetically distinct ‘southeastern’ clade. Although we did not have samples from northeastern Iran or southern Turkmenistan (the known breeding areas of *P*. *c*. *menzbieri*), the presence of a continuous habitat belt from Armenia, along the Elburs mountain range across northern Iran, to Turkmenistan suggests that our southeastern clade represents *P*. *c*. *menzbieri*. Our data indicate that the eastern part of the Lesser Caucasus and southern Armenia represent an introgression zone between *P*. *c*. *caucasicus* and *P*. *c*. *menzbieri*.

#### The Mount Hermon Chiffchaffs

The Anti-Lebanon mountain range has never been included in the breeding range of the Common Chiffchaff [1,4,6,7,16,40]. However seven birds were captured and sampled on Mount Hermon during June, July and August 2013/14 (S1 File) including adults with brood patches. Photographs of these birds will be presented elsewhere, but none of the Mount Hermon birds showed any unusual plumage or biometric characteristics and resembled *P*. *c*. *brevirostris/caucasicus*. Furthermore, both July birds were local fledglings from different localities, confirming a previously undiscovered breeding population. The presence of *P*. *c*. *brevirostris/caucasicus* and *P*. *c*. *collybita* at Mt. Hermon could be explained by recruitment of wintering females from the Caucasus, Anatolia, Rhodope Mountains, or Balkans. Four of the breeding birds had ND2 alleles consistent with this. However, the other three birds had a novel ND2 haplotype and carried some ACO1I9 alleles not found in other chiffchaffs. The level of genetic divergence from other Common Chiffchaff was similar to that which separates the recognized subspecies from each other (Fig 3), suggesting the presence in the Middle East of a new, currently unnamed, Common Chiffchaff taxon. Further work is necessary to resolve the potentially complex biogeographic history of chiffchaffs in this region.

### Taxonomic implications

The results of this study encompass analysis of over 250 chiffchaffs of probably all taxa from multiple localities within their range, and incorporate both nuclear and mitochondrial genes. The data are limited and must be interpreted in light of the need for further genomic analyses and further sampling of more individuals. However, they present the chiffchaffs as a complex of taxa whose divergences can be associated with the dramatic climactic fluctuations and range fragmentations of the last 1 million years, resulting in geographically restricted populations of birds apparently at varying stages of the speciation process. At one extreme, the Mountain Chiffchaff *P*. *sindianus* can now be concluded to be divergent from all other taxa in at least one mitochondrial and one nuclear locus, to show plumage and vocal differences from other chiffchaffs and to overlap broadly in range with some populations of Common Chiffchaff without apparent hybridization or introgression in the Greater Caucasus and Transcaucasia, thereby fulfilling all the criteria of a ‘strict’ biological species. Several authorities treat *P*. *s*. *sindianus* and *P*. *s*. *lorenzii* as separate species (e.g. [3]), but this is not strongly supported by our genetic data. The Iberian Chiffchaff *P*. *ibericus* is morphologically and vocally distinct, divergent from all other taxa at the nuclear and mitochondrial loci, but is parapatric to nominate Common Chiffchaff with a narrow 20–55 km hybrid zone that has been well studied [36]. Hence it seems to be a young, but apparently good, evolutionary species [6]. The Canary Islands Chiffchaff *P*. *canariensis* breeds allopatrically to other chiffchaffs and is vocally distinct. However, although it is probably distinct at mtDNA level (notwithstanding the single bird in this study with nominate *collybita* ND2 sequence), it shares alleles of nuclear loci with the Common Chiffchaff. The Canary Islands Chiffchaff requires further study but its widely accepted status as a full species or an ‘allospecies’ [46] is defensible. It should be noted that the eastern Canary Islands used to have their own taxon, *P*. *canariensis exsul* (now extinct), which may have been more likely to be exposed to introgression with nominate *P*. *collybita*.

The ‘Siberian’ Common Chiffchaff *P*. *c*. *tristis* is morphologically and vocally distinct from other taxa of Common Chiffchaff [6,16] with distinct, unique, mitochondrial haplotypes and a relatively narrow overlap zone with *P*. *c*. *abietinus*, in which intergradation occurs but birds are in part separated by habitat choice [12]. It is broadly distinct at the nuclear locus from other chiffchaff taxa, although some alleles are shared, either through introgression or retention of ancestral shared lineages. The existence of ‘*fulvescens*’ individuals of *P*. *c*. *tristis* east of the Ural that have olive and yellow tones to the mantle and supercilium respectively, characteristics associated with P. *c*. *abietinus*, suggests more widespread introgression. Although further study is merited, the *P*. *c*. *tristis* is arguably at least to be in a stage of incipient speciation or ‘semispecies’ status [46], and valid arguments could be made for either subspecies or species status.

In the southeast of the range of Common Chiffchaff, there are at least two mitochondrial genetic clades of subtly morphologically divergent taxa, *P*. *c*. *brevirostris/caucasicus* and *P*. *c*. *menzbieri*, that intergrade with each other in Transcaucasia and the Lesser Caucasus, and possibly with nominate *P*. *c*. *collybita* and *abietinus* in the Balkans. Although the principal components analysis (Fig 5) placed birds in these locations as a grouping discrete from ‘European’ and ‘Siberian’ Common Chiffchaff, they share ACO1I9 alleles with the other Common Chiffchaff taxa and their treatment as a distinct subspecies group within the Common Chiffchaff is justified.

West European *P*. *c*. *collybita* and east European *P*. *c*. *abietinus*, although genetically distinct, are morphologically extremely similar and, can be distinguished only on mean biometric differences. They co-occur in northeastern Germany, northern and eastern Poland [40], southwestern Ukraine [1], and in Sweden [11], with introgression (Fig 2) suggestive of possible secondary contact after divergence in glacial refugia. The slight but robust mitochondrial genetic differentiation between *P*. *c*. *collybita* and Northern/Eastern European *P*. *c*. *abietinus* is not mirrored by any significant nuclear allele divergence (Figs 4 and 5). Our conservative estimate of the introgression zone breadth between the sister mtDNA clades corresponding to *P*. *c*. *collybita* and *P*. *c*. *abietinus* is 481 km i.e. is almost an order of magnitude greater than those between *P*. *c*. *collybita* and *P*. *ibericus* or between *P*. *c*. *abietinus* and *P*. *c*. *tristis*, which supports their status as (subtly) different subspecies. Whether there are any biological barriers to free gene flow remains to be determined.

The recently discovered breeding population of chiffchaffs on Mt. Hermon is shown here to belong to the Common Chiffchaff *P*. *collybita* and appears to have had a long history. The presence of *P*. *c*. *collybita* and *P*. *c*. *brevirostris/caucasicus* mitochondrial haplotypes in this population would suggest recent establishment of a breeding population by wintering birds from elsewhere, but the coexistence of a novel, highly divergent ND2 sequence is more consistent with a long established relict population. Possibly both alternatives are correct, but further exploring for and sampling of new Common Chiffchaff populations elsewhere in Syria, Israel, Lebanon and southern Turkey would resolve the true status of this novel lineage. The chiffchaff complex is a paradigm model system for ‘speciation in action’ and would repay further studies with more extensive sampling and full genomic analysis.

## Supporting information

S1 File All sampled individuals used in this study with GenBank accession numbers. (XLSX)

S2 File Phylogenetic relationships of mtDNA CytB haplotypes. Bayesian tree representing relationships among CytB clades of Common Chiffchaffs and other chiffchaff species. GenBank accession numbers and inferred subspecies assignment are listed on the right. Numbers next to branches indicate their posterior probability values.
(PDF)
