# Supplementary figures and images for: Geographic patterns of mtDNA and Z-linked sequence variation in the Common Chiffchaff and the ‘chiffchaff complex’
Source: PLoS One. 2019 Jan 4;14(1):e0210268. doi: 10.1371/journal.pone.0210268 (PMC6319743; doi:10.1371/journal.pone.0210268)

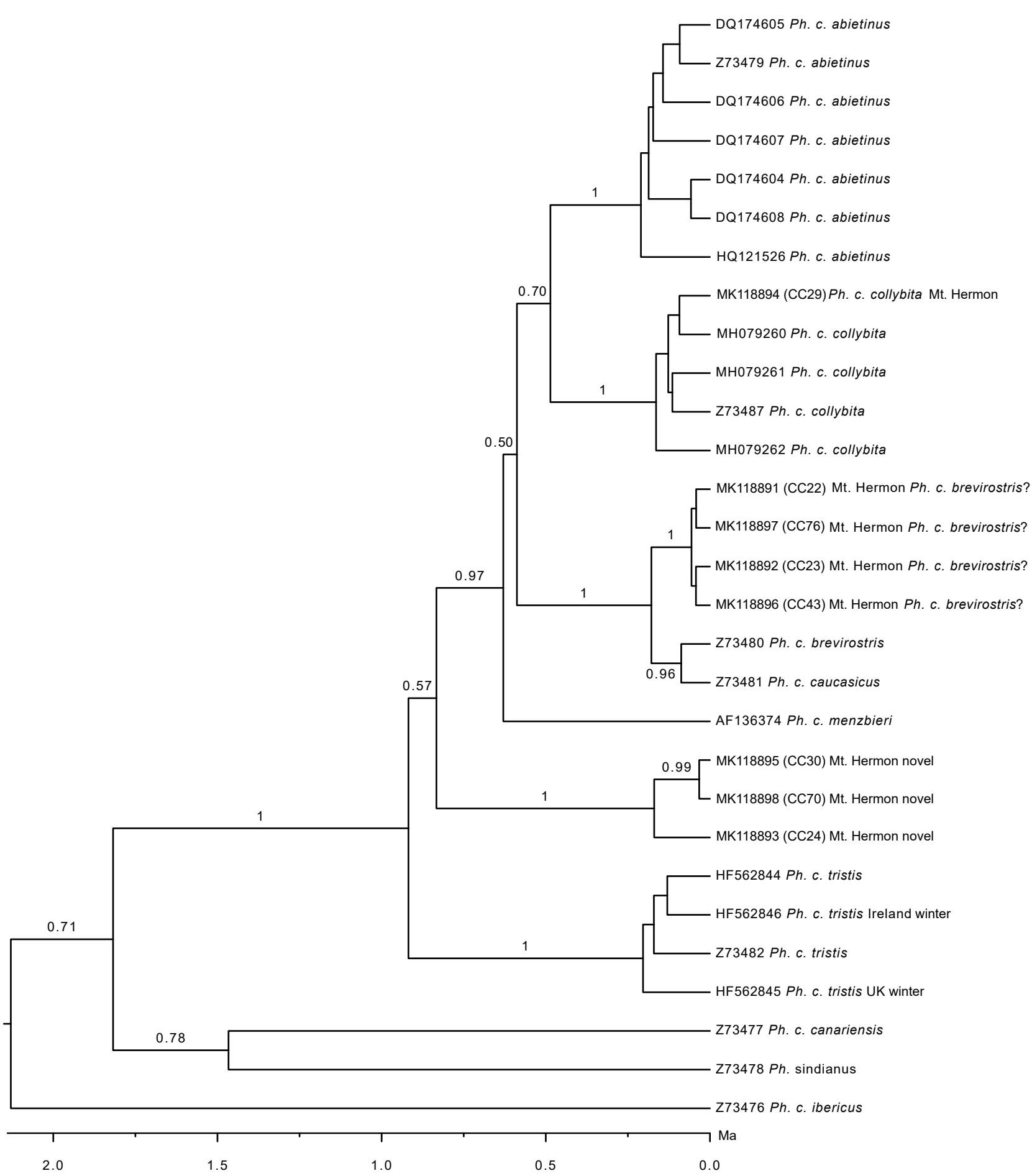

Supplement: S2 File — Bayesian tree representing relationships among CytB clades of Common Chiffchaffs and other chiffchaff species. GenBank accession numbers and inferred subspecies assignment are listed on the right. Numbers next to branches indicate their posterior probability values. (PDF) [file pone.0210268.s002.pdf]
